# ﻿Revision of the Chinese species of the genera *Genimen* and *Genimenoides* (Orthoptera, Acrididae)

**DOI:** 10.3897/zookeys.1251.150870

**Published:** 2025-09-04

**Authors:** Fang-Ting Li, Miao Li, Hong Song, Ben-Yong Mao

**Affiliations:** 1 College of Agriculture and Biological Science, Dali University, Dali, Yunnan 671003, China Dali University Dali China; 2 Co-Innovation Center for Cangshan Mountain and Erhai Lake Integrated Protection and Green Development of Yunnan Province, Dali University, Dali, Yunnan 671003, China Dali University Dali China; 3 Cangshan Forest Ecosystem Observation and Research Station of Yunnan Province, Dali University, Dali, Yunnan 671003, China Dali University Dali China; 4 Research Center for Northwest Yunnan Biodiversity, Dali University, Dali, Yunnan 671003, China Dali University Dali China

**Keywords:** Grasshoppers, identification key, morphology, new combination, new synonym, taxonomy

## Abstract

Based on an examination of types and additional materials, Chinese species in the genera *Genimen* and *Genimenoides* were reviewed. As a result of our study, *Genimen burmanum* Ramme, 1941 was shown not to occur in China, the species *Genimen zhengi* Mao, Ren & Ou, 2010, **syn. nov.** was synonymized under *Genimen yunnanensis* Zheng, Huang & Liu, 1988, and *Genimenoides vittatum* Mao, Ren & Ou, 2010 was transferred to *Genimen*, as the new combination *Genimen vittatum* (Mao, Ren & Ou, 2010), **comb. nov.** An updated species key for *Genimen* is provided.

## ﻿Introduction

The genus *Genimen* was erected by Bolívar in 1917, with *Genimen prasinum* Bolívar I., 1917 from India as its type species. Uvarov (1927) described two species from Sri Lanka, *Genimen ceylonicum* Uvarov, 1927 and *Genimen subapterum* Uvarov, 1927, and the latter was subsequently removed to establish the genus *Genimenoides* by Henry (1934). Since then, *Genimen* has undergone the addition of several species. Ramme (1941) described two species from Myanmar, *Genimen burmanum* Ramme, 1941 and *Genimen victoriae* Ramme, 1941. Zheng et al. (1988) described one species, *Genimen yunnanensis* Zheng, Huang & Liu, 1988. Ingrisch et al. (2004) described two species from India, *Genimen amarpur* Ingrisch, Willemse & Shishodia, 2004, and *Genimen lailad* Ingrisch, Willemse & Shishodia, 2004. Mao et al. (2010) described two species from China, *Genimen bannanum* Mao, Ren & Ou, 2010 and *Genimen zhengi* Mao, Ren & Ou, 2010. Moreover, Zheng (1980) reported a record of *Genimen burmanum* in Yunnan, China, based on a female specimen. However, we have never collected any specimens of *Genimen burmanum* despite our long-term and broad-ranging collections of Acridoidea in Yunnan, so we doubt the authenticity of this species in Yunnan.

The genus *Genimenoides* Henry, 1934 currently contains three species, namely *Genimenoides coloratum* Henry, 1934, *Genimenoides subapterum* (Uvarov, 1927), and *Genimenoides vittatum* Mao, Ren & Ou, 2010, of which the former two are distributed in Sri Lanka. *Genimenoides vittatum* was described based on a single female specimen from Gengma County, Yunnan Province, China, and was placed in *Genimenoides* due to the possession of a scale-like tegmen and reduced tympanum. The original author indicated that the species is similar to *Genimen burmanum* Rammer, 1940 in coloration and appearance, and implied that the discovery of the male of this species might necessitate a revision of its taxonomic status. However, this species is very rare. Since a female was collected in 2004, it has not been found in many subsequent investigations until one male and two females were found in Lancang County in 2023, as well as two males and two females in Ximeng County in 2024. These three counties are adjacent to each other, having subtropical monsoon climates; the maximum linear distance among the three collecting localities is only 138 kilometers. Based on most of the morphological characters of the species, especially the male genitalia, which are more similar to those of *Genimen*, we suggest it should be transferred to *Genimen* as a new combination, *Genimen vittatum* (Mao, Ren & Ou, 2010), comb. nov. to clarify the taxonomy of this group.

## ﻿Material and methods

**Figure 1.**
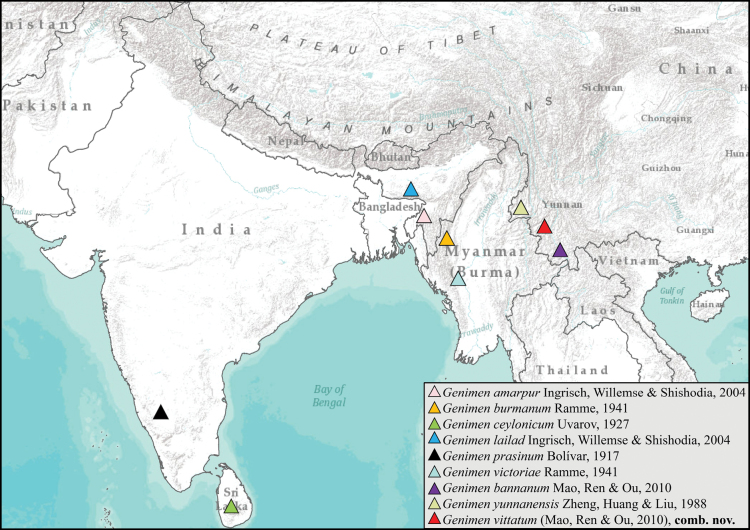
Distribution map of species of *Genimen* Bolívar, 1917.

**Figure 2.**
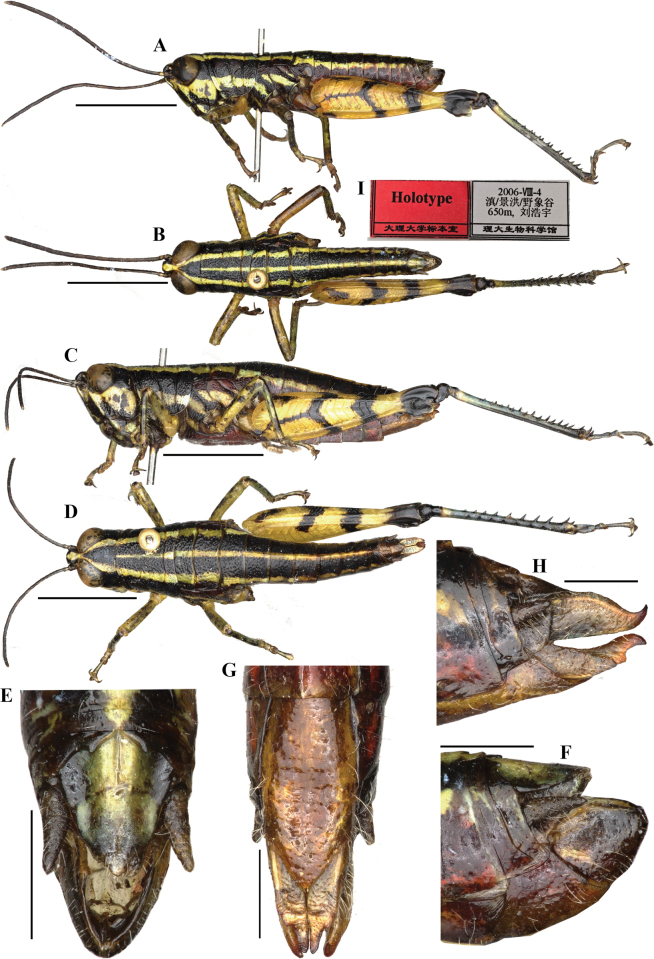
*Genimen bannanum* Mao, Ren & Ou, 2010. A, B. Male habitus, lateral, and dorsal views, respectively; C, D. Female habitus, lateral and dorsal views, respectively; E, F. Male terminalia, dorsal, and lateral views, respectively; G, H. Female terminalia, ventral and lateral views, respectively; I. Holotype label. Scale bars: 5 mm (A–D); 1 mm (E–H).

**Figure 3.**
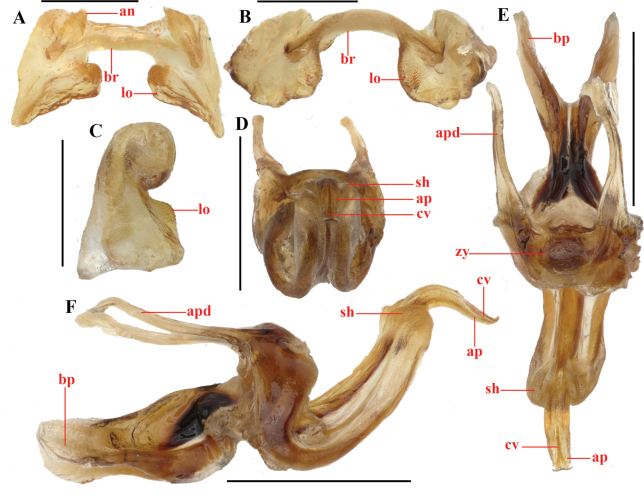
*Genimen bannanum* Mao, Ren & Ou, 2010. A–C. Male epiphallus in dorsal, posterior, and lateral views, respectively; D–F. Phallic complex, apical, dorsal, and lateral views, respectively. Scale bars: 0.5 mm (A–C); 1 mm (D–E). Abbreviations: an = anterior projection, ap = apical valves of penis, apd = apodeme, bp = basal valves of penis, br = bridge, cv = cingular valve, lo = lophi, sh = membranous sheath, zy = zygoma.

**Figure 4.**
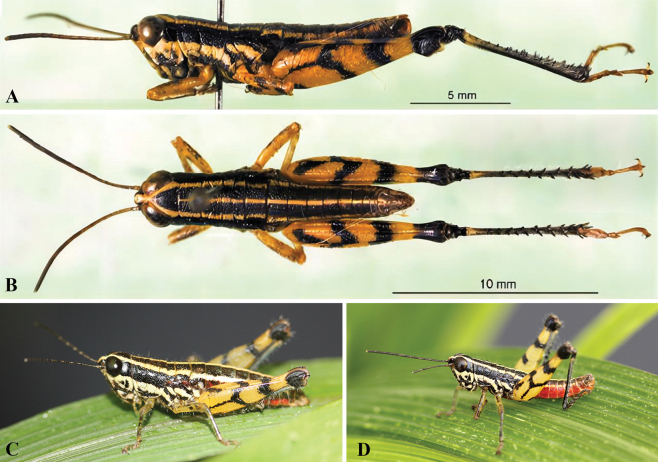
Adult habitus of *Genimen burmanum*. A, B. Male (from http://orthoptera.speciesfile.org/) and *Genimen bannanum*; C, D. Female and male, respectively.

**Figure 5.**
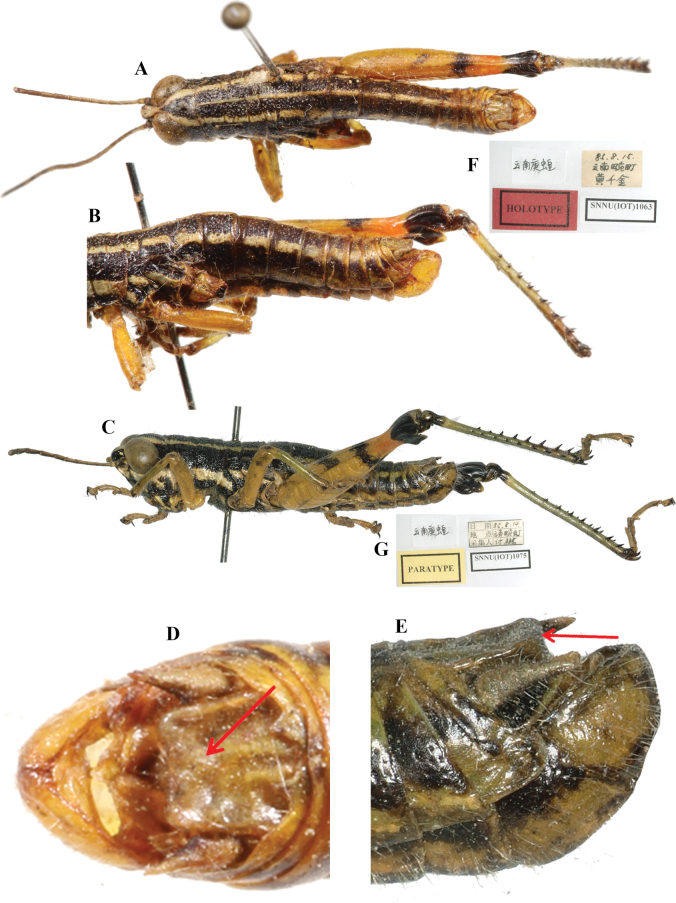
*Genimen yunnanensis* Zheng, Huang & Liu, 1988. A, B. Male, holotype (in IZSNU), dorsal and lateral views, respectively; C. Male, paratype, lateral view; D, E. Male terminaliae of holotype and paratype, dorsal and lateral views, respectively (red arrow indicating the thickening of epiproct); F, G. Holotype and paratype labels, respectively.

**Figure 6.**
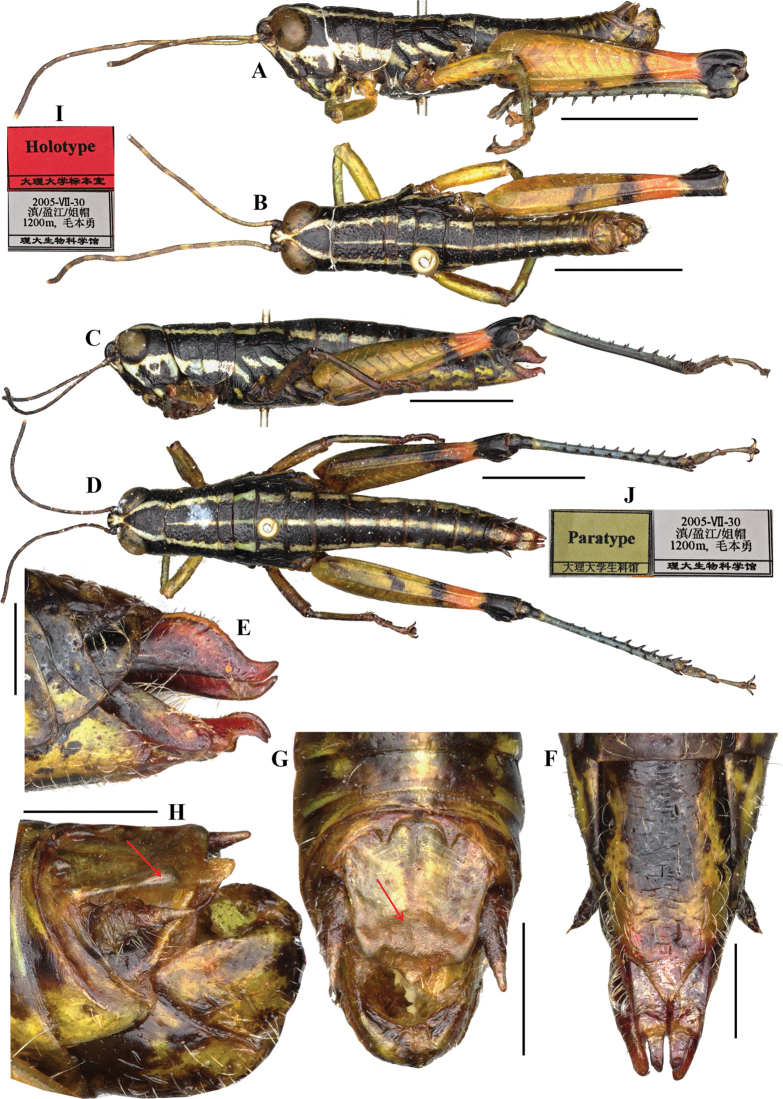
*Genimen zhengi* Mao, Ren & Ou, 2010: 42, syn. nov. = *Genimen yunnanensis* Zheng, Huang & Liu, 1988. A, B. Male holotype habitus in lateral and dorsal views, respectively; C, D. Female paratype habitus in lateral and dorsal views, respectively; E, F. Female terminalia, lateral and ventral views, respectively; G, H. Male terminalia, dorsal and lateral views, respectively (red arrow indicating the thickening of epiproct); I, J. Holotype and paratype labels, respectively. Scale bars: 5 mm (A–D); 1 mm (E–H).

**Figure 7.**
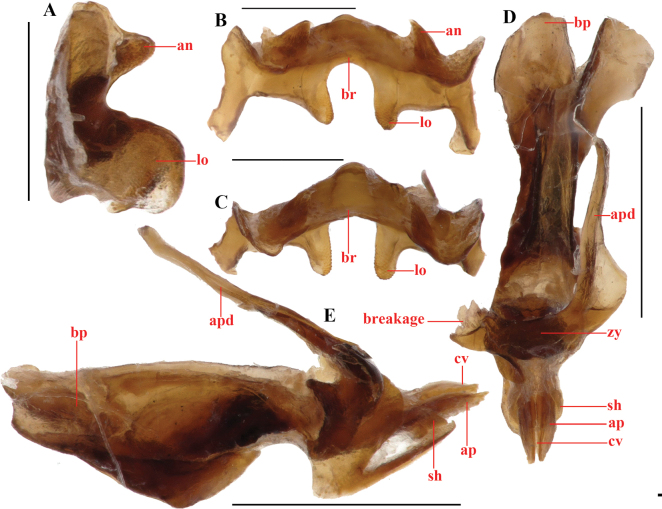
*Genimen zhengi* Mao, Ren & Ou, 2010: 42, syn. nov. = *Genimen yunnanensis* Zheng, Huang & Liu, 1988. A–C. Epiphallus, in lateral, dorsal, and posterior views, respectively; D, E. Phallic complex, in dorsal and lateral views, respectively. Scale bars: 0.5 mm (A–C); 1 mm (D, E). Abbreviations: an = anterior projection, ap = apical valves of penis, apd = apodeme, bp = basal valves of penis, br = bridge, cv = cingular valve, lo = lophi, sh = membranous sheath, zy = zygoma.

**Figure 8.**
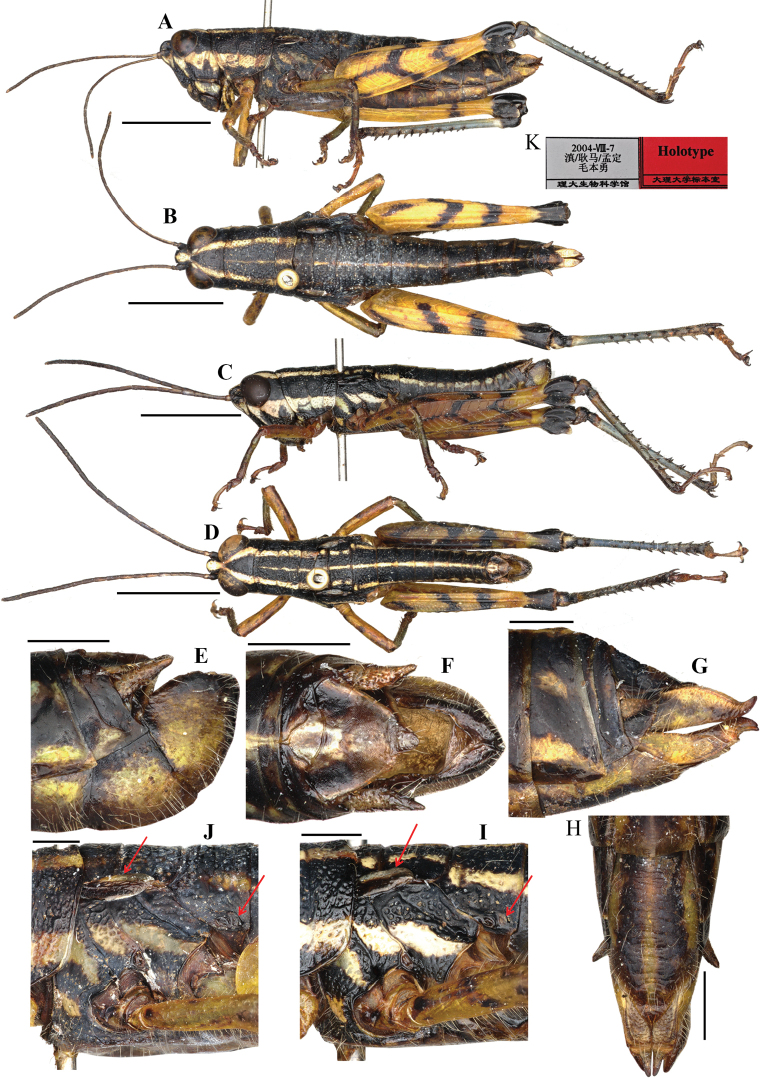
*Genimen vittatum* (Mao, Ren & Ou, 2010), comb. nov. A, B. Female holotype habitus, lateral and dorsal views, respectively; C, D. Male habitus, lateral and dorsal views, respectively; E, F. Male terminalia, lateral and dorsal views, respectively; G, H. Female terminalia, lateral and ventral views, respectively; I, J. Mesothorax, metathorax, and the first tertum of abdomen, male and female, lateral views, respectively (red arrows indicating tegmen and tympanum); K. Holotype labels. Scale bars: 5 mm (A–D); 1 mm (E–J).

**Figure 9.**
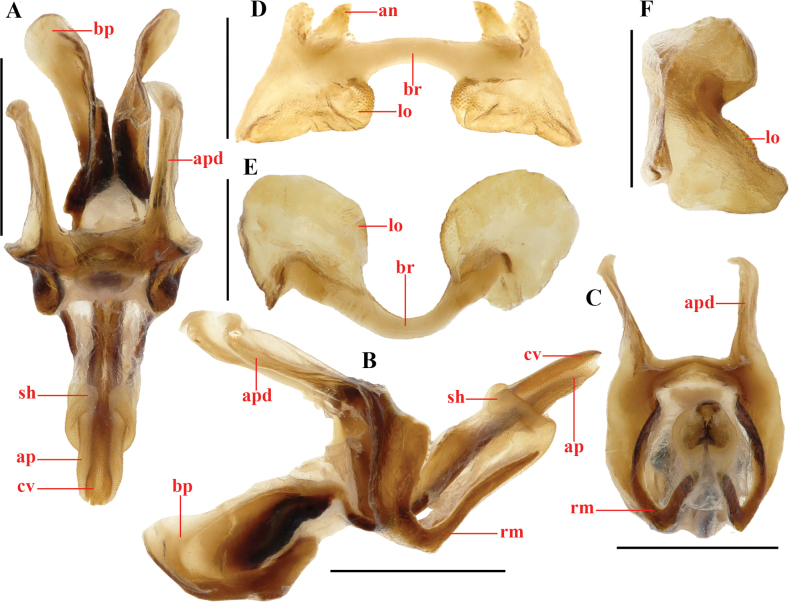
*Genimen vittatum* (Mao, Ren & Ou, 2010), comb. nov. A–C. Phallic complex, in dorsal, lateral, and apical views, respectively; D–F. Epiphallus in dorsal, posterior, and lateral views, respectively. Scale bars: 1 mm (A–C); 0.5 mm (D–F). Abbreviations: an = anterior projection, ap = apical valves of penis, apd = apodeme, bp = basal valves of penis, br = bridge, cv = cingular valve, lo = lophi, rm = rami, sh = membranous sheath, zy = zygoma.

**Figure 10.**
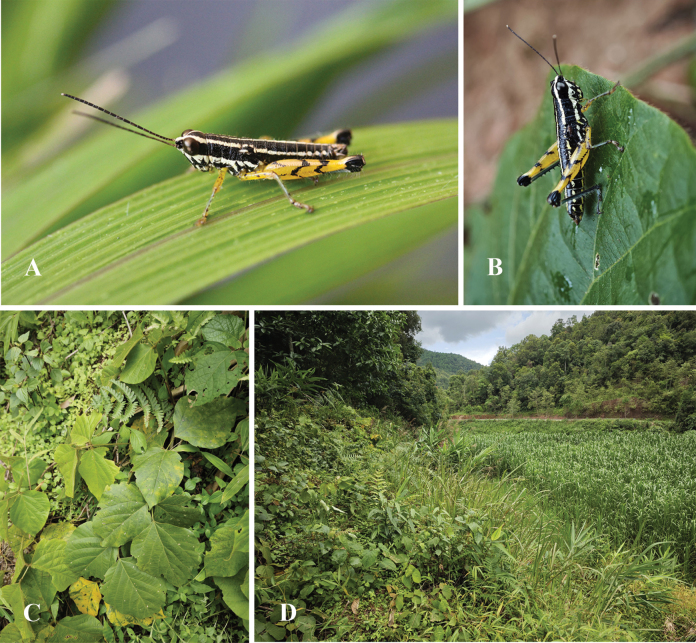
Live adult habitus and habitat of *Genimen vittatum* (Mao, Ren & Ou, 2010), comb. nov. A, B. Male and female, lateral views; C, D. Habitat in Ximeng County, Yunnan Province.

The specimens examined were stored in collections of the Biological Science Museum, Dali University, China (**BMDU**), and the Institute of Zoology, Shaanxi Normal University, Xi’an, China (**IZSNU**). The morphological terminology and measurements followed Uvarov (1966), Dirsh (1975), and Ingrisch et al. (2004). The terminology of male genitalia followed Dirsh (1956). Fig. 1 was made using ArcGIS 10.8, and the map data comes from https://www.tianditu.gov.cn. The color images in Figs 2, 3, 6, 7, 8, 9 were prepared using a digital microscope (Keyence VHX-7000N), and those in Figs 4, 5, 10 were photographed with a Canon digital camera (EOS 60D) or smartphone (Xiaomi 14). The color images in Fig. 5 were made by Mr Hao Tang and Ms Xiongyan Yin. The final illustrations were prepared as plates using Adobe Photoshop® CS2 software.

## ﻿Taxonomy

### ﻿ Genimen

Taxon classificationAnimaliaOrthopteraAcrididae

Bolívar, 1917

11E35B45-7731-55BF-A8F4-19A3943C38A9

Genimen Bolívar, 1917: 401; Henry 1934: 193; Willemse 1957: 17, 342; Zheng and Shi 1998: 163; Yin et al. 1996: 303; Li et al. 2006: 399; Mao et al. 2010: 40.

#### Type species.

*Genimen prasinum* Bolívar, 1917.

#### Generic diagnosis.

Body size small. Head shorter than or equal to pronotum. Frons slightly oblique in lateral view, frontal ridge projecting between antennae, obsolete below the transverse facial furrow. Fastigium somewhat projecting; interocular distance smaller than the width of the antennal scape. Eyes protruding, nearly rounded. Antennae filiform, slender, and elongate, exceeding the posterior margin of pronotum. Pronotum cylindrical, with prozona strongly longer than metazona; median carinae not elevated; lateral carinae absent. Prosternal tubercle short, apex conical. Mesosternal lobes wider than long; metasternal lobes contiguous in male or separate in female. Hind femur moderately thick, upper carina smooth, lower genicular lobes rounded apically; hind tibia without dorso-external apical spine. Apterous or micropterous. Tympanum distinct or reduced. Male terminalia: tenth tergite with or without furculae; epiproct triangular or shield-shaped; cercus conical; subgenital plate short and conical, apex obtuse; epiphallus with bridge arched, not divided in the middle; phallic complex with apical penis valves prolonged, sheathed by a membranous sheath. Female terminalia: epiproct triangular; cercus conical; valves of ovipositor short, nearly smooth, apices hooked.

#### Composition and distribution.

Nine species are currently assigned to the genus *Genimen*, including *Genimen amarpur* Ingrisch, Willemse & Shishodia, 2004, *Genimen bannanum* Mao, Ren & Ou, 2010, *Genimen burmanum* Ramme, 1941, *Genimen ceylonicum* Uvarov, 1927, *Genimen lailad* Ingrisch, Willemse & Shishodia, 2004, *Genimen prasinum* Bolívar, 1917, *Genimen victoriae* Ramme, 1941, *Genimen yunnanensis* Zheng, Huang & Liu, 1988, and *Genimen zhengi* Mao, Ren & Ou, 2010. In this paper, we propose a new synonym, *Genimen zhengi* Mao, Ren & Ou, 2010, syn. nov. under *Genimen yunnanensis* Zheng, Huang & Liu, 1988 and a new combination, *Genimen vittatum* (Mao, Ren & Ou, 2010), comb. nov. Thus, a total of nine species are distributed in the Oriental Realm, among them three species in India, one species in Sri Lanka, two species in Myanmar, and three species in China (Fig. 1).

### ﻿Key to the known species of *Genimen*

**Table:** 

1	Tegmina absent; tympana big, distinct	**2**
–	Tegmina small, laterally positioned, reaching the middle of the metanotum; tympana obsolete, surface somewhat sclerotized, margin blurry	***Genimen vittatum* (Mao, Ren & Ou, 2010), comb. nov.**
2	Dorsum of body without longitudinal stripe	**3**
–	Dorsum of body with three longitudinal stripes	**4**
3	Median carina on dorsum of pronotum and abdomen noticeable; postocular bands extending to the end of abdomen; hind femora without Y-shaped black spots in the middle of the outer surface	***Genimen prasinum* Bolívar, 1917**
–	Median carina on dorsum of pronotum and abdomen faint; postocular bands extending to the first abdominal tergite; hind femora with Y-shaped black spots in the middle of the outer surface	***Genimen victoriae* Ramme, 1941**
4	Hind femora without Y-shaped or V-shaped black marking on the external area	**5**
–	Hind femora with clear Y-shaped or V-shaped black marking on the external area	**7**
5	Hind femora yellowish green and without black spots	***Genimen ceylonicum* Uvarov, 1927**
–	Hind femora yellow and with a middle black ring in the apical half, dorsal area with a black marking in the middle	**6**
6	Pronotum and abdominal tergites with three longitudinal white stripes dorsally; hind femora with orange-red preapical ring; male 10^th^ abdominal tergite separated in the middle	***Genimen yunnanensis* Zheng, Huang & Liu, 1988**
–	Pronotum and abdominal tergites with three longitudinal yellow stripes dorsally; hind femora without preapical ring; male 10^th^ abdominal tergite not separated in the middle	***Genimen lailad* Ingrisch, Willemse & Shishodia, 2004**
7	Hind femora with V-shaped black stripe on the outer surface in males or Y-shaped black stripe in females; abdomen laterally yellow; female mesosternal interspace narrower than mesosternal lobes	***Genimen amarpur* Ingrisch, Willemse & Shishodia, 2004**
–	Hind femora with Y-shaped black stripes on the outer surface in both sexes; abdomen laterally black or reddish brown; female mesosternal interspace wider than mesosternal lobes	**8**
8	Upper side of hind femur with a larger black transverse spot before the middle, which connects with the Y-shaped black stripe on the outer surface; abdomen laterally black in male or reddish brown in female; furculae on posterior margin of male 10^th^ abdominal tergite indistinct	***Genimen burmanum* Ramme, 1941**
–	Upper side of hind femur with smaller black bands before the middle, which separate from the Y-shaped black stripe on the outer surface; abdomen laterally black, staining red in both sexes; furculae on posterior margin of male 10^th^ abdominal tergite distinct	***Genimen bannanum* Mao, Ren & Ou, 2010**

### ﻿Taxonomy of Chinese species of *Genimen*

#### ﻿ Genimen bannanum

Taxon classificationAnimaliaOrthopteraAcrididae

Mao, Ren & Ou, 2010

20DF57DB-B03F-5D1C-898E-577B5CCC9B76

Figs 2, 3, 4 Chinese common name: 版纳庚蝗

Genimen bannanum Mao, Ren & Ou, 2010: 40 (holotype—male, China: Yunnan: Xishuangbanna, in BMDU; examined).
Genimen burmanum Ramme, 1941; Zheng 1980: 130 (1 female, China: Yunnan, Mengla) [Misidentification].

##### Type material examined.

***Holotype*** • ♂, China: Yunnan, Xishuangbanna, alt. 850 m, 4 August 2006, deposited in BMDU.

##### Other material examined.

• 2♀, China: Yunnan, Menghai, 22°13′N, 100°37′E, alt. 837 m, 27 Jul. 2023, leg. Zhilong Yin. • 1♂1♀, China: Yunnan, Menglian, 10 May. 2023, leg. Zhilong Yin. • 2♂, China: Yunnan, Jinghong, 25 Jul. 2013, leg. Benyong Mao.

##### Differential diagnosis.

*Genimen bannanum* is closely related to *Genimen burmanum* as demonstrated by the pronotum and abdomen with three longitudinal yellow stripes on the dorsum, and by the hind femora with a Y-shaped black marking on the outer side (Fig. 2A–D, 4A, B). However, it can be distinguished from the latter by the following characters: male 10^th^ abdominal tergite with clear triangular furculae on posterior margin (Fig. 2E, F) (the furculae being indistinct in the latter (Fig. 4B)); upper side of hind femur with a short black transverse spot before the middle, which separate from the Y-shaped mark of the outer side (Figs 2A–D, 4C, D) (the black transverse spot stout and connected with Y-shaped mark in *Genimen burmanum* (Fig. 4A, B)). Moreover, in *Genimen bannanum* the phallic organ possesses following features: bridge of epiphallus strongly narrow, slightly arched in dorsal view; lophi roughly parallelogrammic, projecting in a more than 90° angle from bridge, with undulating thick margins and obtuse inner angle; anchorae large, compressed, apex subacute, pointing apically (Fig. 3A–C); apical penis valves of the phallic complex strongly prolonged, in which the basal two-thirds being sheathed by a very long membranous sheath and the apical third upcurved, distally tapering as a beak-shaped in lateral view (Fig. 3D–F).

##### Remarks.

Ramme (1941) described the species *Genimen burmanum* from Dudan Taung and Hmaubi, Myanmar, two localities near Yangon. However, he only described the body color, not the morphological characteristics. Zheng (1980) reported the new distribution of *Genimen burmanum* in China, based on female specimens collected from Mengla County, Yunnan Province. Li et al. (2006) agreed with the new distribution and described the first male morphological characters, quoting the female data of Zheng (1980) instead of the male specimen. However, we have never collected any specimens of *Genimen burmanum* despite the long-term and broad-range collections of Acridoidea in Yunnan, so we doubt the authenticity of this species in Yunnan.

Zheng’s (1980) drawing of the hind femur, showing that the black transverse spot located before the middle on the upper side is separate from the Y-shaped mark on the outer side, agrees with *Genimen bannanum* rather than *Genimen burmanum*. Zheng’s (1980) other descriptions for the nominal *Genimen burmanum* agree with the female of *Genimen bannanum*. Therefore, the conclusion that “*Genimen burmanum* being distributed in Yunnan” is based on the misidentification of *Genimen bannanum* and is, thus, unreliable. As a result of our study, *Genimen burmanum* was shown not to occur in China.

#### ﻿ Genimen yunnanensis

Taxon classificationAnimaliaOrthopteraAcrididae

Zheng, Huang & Liu, 1988

60067A03-E3C6-5A2B-A580-C0BB1A31360D

Figs 5, 6, 7 Chinese common name: 云南庚蝗

Genimen yunnanensis Zheng, Huang & Liu, 1988: 83 (holotype—male, China: Yunnan: Ruili (Wanding), in IZSNU; examined).
Genimen zhengi Mao, Ren & Ou, 2010, syn. nov.: 42.

##### Type material examined.

*Genimen yunnanensis*, • ***Holotype*** ♂, ***Allotype*** ♀, ***Paratype*** 6♂4♀, China: Yunnan, Ruili, Wanding, 14–15 August 1985, in IZSNU. • *Genimen zhengi*, ***Holotype*** ♂, ***Paratype*** 1♂1♀, China: Yunnan, Yinjiang, Jiemao, 30 July 2005, in BMDU.

##### Additional material.

*Genimen yunnanensis*, • 2♀, China: Yunnan, Yingjiang, Nabang, 31 July 2009, in BMDU; • 1♀, China: Yunnan, Yingjiang, Nabang, 21 August 2023, in BMDU.

##### Redescription.

Body small; surface with scattered or dense punctures (Figs 5A–C, 6A–D). Head conical, nearly as long as pronotum; fastigium small, roundly protruding; interocular distance extremely narrow, about 0.4 (♂) or 0.5 (♀) times as wide as frontal ridge between antennae, and about 0.6 (♂) or 0.8 (♀) times as wide as scape of antennae; face oblique in profile; frontal ridge projecting between antennae, absent below the transverse facial furrow, with shalow longitudinal sulcus below antennae; lateral facial keels straight; subocular furrow nearly obsolete. Antennae filiform, reaching the base of the hind femur (♂) or surpassing the posterior margin of the pronotum (♀), median segments about 2.0–3.0 (♂) or 3.3 (♀) times longer than wide. Eyes protruding, nearly rounded, longitudinal diameter about 1.2–1.4 (♂) or 1.2 (♀) times as long as horizontal diameter, and about 1.8–1.9 (♂) or 1.6 (♀) times longer than subocular furrow. Pronotum nearly cylindrical; anterior margin straight, or weakly concave in the middle, posterior margin shallowly concave; median carina indicated by a smooth line, not elevated, interrupted by last sulcus only; lateral carinae absent; prozona with scattered punctures, metazona with dense ones; prozona 4.0–4.3 (♂) or 4.0 (♀) times as long as metazona (Fig. 6A–D). Prosternum as a whole bulging; prosternal spine small, conical, and apex subacute. Mesosternal lobes 1.2–1.4 (♂) or 1.2 (♀) times wider than long; mesosternal interspace about 1.4–1.8 (♂) or 1.1 (♀) times longer than minimal width; metasternal lobes contiguous (♂) or separate (♀). Apterous (Figs 5A–C, 6A–D). Hind femora with upper carina smooth; lower knee lobes with apex rounded; hind tibiae cylindrical, with 8 external and 9 internal spines on dorsal side in both sexes; external apical spine absent; length of hind tarsi nearly as long as half of hind tibiae, length of 3^rd^ tarsomere as long as that of 1^st^ and 2^nd^ tarsomeres together (Figs 5A–C, 6A–D). Abdomen with median carina smooth. Tympana slightly reduced but clear, oval, about twice as large as the anterior stigma, surface somewhat sclerotized (Figs 5B, 6A). Male 10^th^ abdominal tergite separate but nearly contiguous in the middle; posterior margin with triangular furculae, roundly concave between furculae (Figs 5D, 6G). Epiproct shield-shaped, basal third with low median longitudinal furrow; lateral areas a little concave; apical third thickened and apex decurved; lateral margins a little constricted in the middle; posterior margin angularly or roundly projecting in the middle (Figs 5D, 6G). Cerci conical, basal half gradually narrowed and apical half sharply tapered, apex subacute, surpassing apex of epiproct (Figs 5D, E, 6G, H). Subgenital plate short, conical, apex obtuse (Figs 5D, E, 6G, H). Epiphallus with bridge arched in dorsal view; lophi projecting at a 90° angle from bridge, weakly incurved; anchorae large, compressed, apex subacute, pointing apically; lateral plate with external margin weakly concave (Fig. 7B, C). Phallic complex: basal penis valves very prolonged and apically expanded; apical penis valves shorter and straight, in which basal 2/3 sheathed by a membranous sheath and apex acute; apodemes with apex not reaching the apex of basal penis valves; zygoma nearly straight or finely arched (Fig. 7D, E).

Female 10^th^ abdominal tergite entire in the middle. Epiproct triangular. Cerci conical. Subgenital plate oblong, posterior margin triangular. Valves of ovipositor hook-like, margins smooth (Fig. 6E, F).

##### Coloration.

(Fig. 6). Body black with three yellowish-white stripes in dorsal view, the median one thinner from the anterior margin of the pronotum to the 10^th^ abdominal tergite. Yellowish-white marking of fastigium separated between the eyes, continuing as lateral bands on the occiput, pronotum, mesothorax, metathorax, and abdomen. Postocular bands black, extending to the abdominal apices. In profile, longitudinal stripe under the eye yellowish white, extending to the pronotum, mesothorax, and metathorax. Antennae brownish black, darker towards the apex, apical segment yellow. Eyes brown. Fore and mid legs with femora and tarsi yellow, and tibiae yellowish green. Hind femora yellow with a broad orange ring before the knee and a narrow black ring before the orange ring, upper side with a black spot in the middle, and knee black. Hind tibiae dark blue with the basal part black and next to a dark yellow ring. Abdominal sternites and terminalia dark yellow with black spots.

##### Measurements (mm).

Length of body: ♂ 13.5–15.0, ♀ 18.5–20.0; length of pronotum: ♂ 3.0–3.5, ♀ 4.0–5.0; length of hind femur: ♂ 7.5–8.0, ♀ 10.0–10.5.

##### Remarks.

We examined the type specimens of *Genimen yunnanensis* and found that its male epiproct was thickened and decurved in the apical third. The posterior margin of the 10^th^ abdominal tergite was roundly concave between furculae (Fig. 5D, E). Both *Genimen yunnanensis* and *Genimen zhengi* share the same morphological characters and are surely conspecific. Therefore, *Genimen zhengi* syn. nov. is regarded as a new junior synonym of *Genimen yunnanensis*. Moreover, we re-described *Genimen yunnanensis* to supplement the brief original description in Chinese.

#### ﻿ Genimen vittatum

Taxon classificationAnimaliaOrthopteraAcrididae

(Mao, Ren & Ou, 2010) comb. nov.

89B15EE7-AD11-5FD4-8B0C-316BB9F97EF1

Figs 8, 9, 10 Chinese common name: 条纹庚蝗

Genimenoides vittatum Mao, Ren & Ou, 2010: 38 (holotype—female, China: Yunnan: Gengma, in BMDU; examined).

##### Material examined.

*Genimenoides vittatum*, ***Holotype***: • ♀, China: Yunnan, Gengma, 22°33′N, 99°4′E, 7 Aug. 2004, leg. Benyong Mao.

##### Other material examined:

• 1♂2♀, China: LYunnan, Lancang, 11 Jun. 2023, leg. Zhilong Yin. • 2♂2♀, China: Yunnan, Ximeng, 19 Jul. 2024, leg. Fingting Li, Hong Song, Xun Wang, Honglei Yu.

##### Distribution.

China: Yunnan.

##### Redescription

(Fig. 8). Body size small, slightly stouter in females. Head conical, distinctly shorter than pronotum; fastigium roundly protruding, width in front of the eyes almost equal to or slightly wider than long; interocular distance narrow, about 0.4 (♂) or 0.5–0.6 (♀) times the width of frontal ridge between antennae; face oblique in lateral view; frontal ridge projecting and broad between antennae, below antennae gradually concave, constricted above the transverse facial furrow and obsolete below it; lateral facial keels straight (Fig. 8A–D). Antennae filiform, reaching the coxa of the hind leg, any median segment about 2.5–2.9 (♂) or 2.3–3.0 (♀) times longer than wide (Fig. 8A–D). Eyes prominent, nearly rounded, longitudinal diameter about 1.2–1.3 (♂) or 1.3 (♀) times as long as horizontal diameter, about 1.5–1.8 (♂) or 1.4–1.5 (♀) times longer than subocular furrow (Fig. 8A–D). Pronotum nearly cylindrical; anterior margin feebly convex, posterior margin shallowly concave; median carina indicated by a smooth line, not elevated, only interrupted by last sulcus; lateral carinae absent; prozona with sparse punctures, metazona with dense punctures; prozona 2.9–3.3 (♂) or 2.9–3.4 (♀) times as long as metazona (Fig. 8A–D). Prosternum as a whole bulging; prosternal spine small, conical, and apex subacute. Mesosternal lobes nearly square; mesosternal interspace about 0.7 (♂) or 0.6 (♀) times longer than minimum width; metasternal lobes nearly contiguous. Tegmina scale-like, small, laterally positioned, surpassing the middle of the metanotum, 1.8–2.5 (♂) or 1.8–2.3 (♀) times longer than maximum width (Fig. 8I, J). Hind femora with upper carina smooth; lower knee lobes with apex roundly angular; hind tibiae cylindrical, with 8 external and 9 internal spines on dorsal side; external apical spine absent; length of hind tarsi about as long as half of hind tibiae, 3^rd^ tarsomere as long as 1^st^ and 2^nd^ tarsomeres together (Fig. 8A–D). Abdomen with median carina smooth. Tympana obsolete, surface somewhat sclerotized, margin blurry, opening a little larger than stigma (Fig. 8I, J). Male 10^th^ abdominal tergite narrowly separated in the middle, posterior margin with small triangular furculae. Epiproct triangular, posterior margin triangularly protruding, 1.0–1.1 times longer than basal maximum width; basal half with shallow median longitudinal sulcus, lateral areas concave (Fig. 8E, F). Cerci conical, basal half gradually narrowed and apical half sharply tapered, apex subacute, surpassing apex of epiproct (Fig. 8E, F). Epiphallus with bridge strongly narrowed, almost straight in dorsal view; lophi enlarged, surface granular, nearly rounded in posterior view, edge thickening, projecting at more than 90° angle from bridge; ancorae larger, subconical (Fig. 9D, F). Phallic complex: basal penis valves about as long as apical penis valves and apically expanded; apical penis valves strongly prolonged, straight in lateral view, apically acute; basal two-thirds of apical penis valves sheathed by a very long membranous sheath; cingular valves fused in ventral side, partially surrounded by apical valves of penis; zygoma straight; rami U-shaped (Fig. 9A–C).

Female 10^th^ abdominal tergite entire in the middle. Supra anal plate triangular. Cerci conical. Subgenital plate with a posterior triangular margin. Valves of ovipositor hook-like, margins smooth (Fig. 8G, H).

##### Coloration.

(Figs 8A–D, 10A, B). Body black with three yellowish-white stripes in dorsal view, the median one thinner from the anterior margin of pronotum to the epiproct. Yellowish-white marking of fastigium separated between the eyes, continuing as lateral bands on the occiput, pronotum, mesothorax, metathorax, and abdomen. Postocular bands black, extending to the abdominal apices. In profile, the yellowish-white longitudinal stripe under the eyes extends to pronotum, mesothorax, and metathorax. Antennae brownish black. Eyes brown. Fore and mid legs with femora and tarsi yellow, tibiae yellowish green. Hind femora yellow with a narrow black ring before the knee; upper side with two black spots in the middle and near the base; outer side with a Y-shaped black mark in the basal half; knee black. Hind tibiae dark blue with the basal part black, next to a yellow ring. Abdominal tergites black with a narrow medial and broader lateral longitudinal yellowish-white stripes; abdominal sternites and terminalia dark yellow with black spots.

##### Measurements (mm).

Length of body: ♂ 16.5–16.7, ♀ 19.0–22.6. Length of pronotum: ♂ 2.8–3.1, ♀ 3.5–3.6. Length of hind femur: ♂ 9.1–9.2, ♀ 9.7–10.9.

##### Remarks.

Due to the possession of a scale-like tegmen and reduced tympanum, the species was previously placed in *Genimenoides*. With the discovery of male individuals, the differences between this species and other species of *Genimenoides* are revealed: 1) body slender (body stout in *Genimenoides*); 2) hind femora slender, outer side not strongly convex (hind femora very stout, outer side strongly convex in *Genimenoides*); 3) eyes smaller, broadly separated dorsally (eyes larger, very narrowly separated dorsally in *Genimenoides*); 4) male cerci without any tooth on the inner side near the apex (in *Genimenoides* male cerci with a small tooth on the inner side near the apex); and 5) female valves of ovipositor with margins smooth (denticulate in *Genimenoides*). Meanwhile, the main morphological features of *Genimenoides vittatum* are more similar to *Genimen*: 1) body slender; 2) narrower interocular distance; 3) male cerci without any tooth on the inner side near the apex; 4) valves of ovipositor with margins smooth; and 5) similar male genitalia with penis valves strongly prolonged and apical penis valves sheathed by membranous sheath. Therefore, it was transferred to *Genimen* as a new combination, *Genimen vittatum* (Mao, Ren & Ou, 2010), comb. nov.

## ﻿Discussion

So far, the two genera *Genimen* and *Genimenoides* have been known only to be distributed in the Oriental region. These species, which are apterous or micropterous and therefore unable to fly, have a very narrow distribution area. The three species living in Yunnan have limited distributions in southern or southwestern Yunnan, where several large rivers, deeply incised valleys, and intermountain lowlands ecologically isolate mountain habitats. Our long-term research and investigations have found that human activities are more frequent near their habitats, threatening their survival (Fig. 10). Therefore, the survival of the *Genimen* species is under great pressure and requires more attention and protection.

The genus *Genimen* is arranged in the tribe Podismini Jacobson, 1905 of the subfamily Melanoplinae, while the genus *Genimenoides* is in an uncertain tribe of the subfamily Catantopinae (Cigliano et al. 2025). The phylogenetic relationship between them and the taxonomic status of *Genimenoides* need to be further studied using phylogenetic topological relations based on molecular and morphological data, especially male genitalia. Unfortunately, due to the rarity of these species, we have not been able to obtain all the necessary samples to carry out systematic research.

## Supplementary Material

XML Treatment for Genimen

XML Treatment for Genimen bannanum

XML Treatment for Genimen yunnanensis

XML Treatment for Genimen vittatum

## ﻿Additional information

### Use of AI

No use of AI was reported.

### Author ORCIDs

Fang-Ting Li https://orcid.org/0009-0006-2682-2696

Miao Li https://orcid.org/0000-0002-4903-1102

Hong Song https://orcid.org/0000-0002-2486-8889

Ben-Yong Mao https://orcid.org/0000-0002-0245-8950

### Data availability

All of the data that support the findings of this study are available in the main text.
